# Coexistence of memory resistance and memory capacitance in TiO_2_ solid-state devices

**DOI:** 10.1186/1556-276X-9-552

**Published:** 2014-10-04

**Authors:** Iulia Salaoru, Qingjiang Li, Ali Khiat, Themistoklis Prodromakis

**Affiliations:** 1 Nano Research Group, School of Electronics and Computer Science, University of Southampton, Southampton SO17 1BJ, UK; 2 College of Electronic Science and Engineering, National University of Defence Technology, Changsha 410073, China

**Keywords:** ReRAM, Memristor, Memcapacitor, TiO_2_, Nanoscale

## Abstract

This work exploits the coexistence of both resistance and capacitance memory effects in TiO_2_-based two-terminal cells. Our Pt/TiO_2_/TiO_*x*_/Pt devices exhibit an interesting combination of hysteresis and non-zero crossing in their current-voltage (*I*-*V*) characteristic that indicates the presence of capacitive states. Our experimental results demonstrate that both resistance and capacitance states can be simultaneously set via either voltage cycling and/or voltage pulses. We argue that these state modulations occur due to bias-induced reduction of the TiO_*x*_ active layer via the displacement of ionic species.

## Background

The memristor (short for memory-resistor) was theoretically conceived in 1971 by Chua [1] through his famous symmetry argument. Even though the first experimental results on resistive memory have been reported as early as in the 1960s [2-5], it did not attract at that time the attention of the scientific community. Nonetheless, after the announcement in 2008 [6] that the missing memristor has been found by researchers at the Hewlett-Packard laboratory, the memristor is put again in the picture. Furthermore, in 2009, Di Ventra et al. [7,8], advanced the field by theoretically defining two other types of devices: memory capacitors (memcapacitors) and meminductors that can be considered as mem-devices [9-11]. To date, concurrent resistive and capacitive switching effect have been observed on practical devices, in perovskite oxide [12], LaAlO_3_[13] and TiO_2_[14,15]. This further leads to a reconsideration of the existing memristor theory; a plausible extension incorporates nano-battery effect. In addition, recently, the research community has shown great interest on exploiting the coexistence of distinct memory modalities in developing adaptive circuits that operate in radio frequencies (RFs) [16].

To date, a large number of materials have been exploited including binary metal oxides, manganite, amorphous Si, doped Si, perovskite oxides and even organic materials [17-26]. Resistive switching has been observed in all these materials and depending on the material employed, distinct mechanisms have been proposed to be causing this resistive change, including the formation and rupture of conductive filaments [27-31] the modulation of Schottky barriers [7,8], electrical trap-related processes [9] and phase change [10] of the active material. Moreover, it was recently demonstrated that the memristor exhibits capacitive memory as well [11-15] that augments the interest of research and industrial communities by introducing novel functionalities and thus applications; beyond what was previously proposed for memristors, i.e. reconfiguration architectures [32], neuromorphic computing [33] and artificial synapses [34].

In this paper, we provide experimental evidence of the coexistence of both resistive and capacitive memory effects in TiO_2_-based nanoscale devices. We present a complete suite of electrical characterisation via quasi-static direct current (DC) voltage sweep, sweeping potentials of static/dynamic frequencies of alternating current (AC) and voltage pulsing. The results demonstrate the concurrent resistive and capacitive switching behaviours in our solid-state prototypes with the effective resistive and capacitive states modulated simultaneously by appropriate voltage pulses. We argue that this effect is related to a bias-induced reduction of the TiO_*x*_ active layer via the displacement of ionic species.

## Methods

### Fabrication of TiO_2_-based active cells

All two terminal devices investigated in this work were fabricated on 4-in. silicon wafers. A 200 nm thick SiO_2_ film was thermally grown on Si wafer to insulate the base layer of the devices. Then, bottom electrodes (BE) were defined by conventional optical lithography using double-layer photoresists and electron-beam evaporation of the 5 nm Ti film and 30 nm Pt film, followed by the lift-off process. A bilayer active core composed of TiO_2_ and oxygen-rich TiO_*x*_ was deposited and patterned on top of the BEs via RF sputtering and photolithography. RF sputtering was performed using a stoichiometric TiO_2_ target and a power of 300 W; the gas flow for TiO_2_ is 30 sccm of Ar and for TiO_*x*_ was 2 sccm of Ar and 10 sccm of O_2_. Finally, a 30 nm thick top electrode (TE) of the Pt film was deposited and patterned following the same method adopted for the BEs. This composition of titanium dioxide was previously studied in [35,36].

### Electrical measurements

All devices were electrically evaluated on wafer via a semiconductor characterisation suite Keithley SCS-4200 (Keithley, Cleveland, OH, USA). During the measurements, the voltage bias was applied to the TE, while maintaining the BE grounded. The probe/point contacts to the TE and BE of the devices under test were realized through a pair of Wentworth probe needles, using a Wentworth Laboratories AVT 702 semi-automatic prober (Brookfield, CT, USA). In order to avoid any problem induced by crossbar devices, such as current sneak paths, all tested devices have stand-alone architecture. The current-voltage (*I*-*V*) characteristics were studied by quasi-static voltage sweeping measurements. The capacitance-voltage (C-V) measurements were carried out using C-V units (CVU) from the same equipment with the measurements frequency ranging from 100 kHz to 1 MHz. In addition, voltage pulsing mode was used to program the devices in different resistive and capacitive states. Several voltage pulses were applied to the device using the two-pulsed measure units (PMUs) of the same characterization system. Voltage pulses of ±6 V of magnitude and duration of 1 ms were used for programming the devices with a small voltage pulse with 0.5 V amplitude and 1 ms pulse width for reading the resistive states. The capacitive states of the devices were measured by employing a small AC 30 mV stimulus at 1 MHz (DC bias 0.5 V).

### Modelling and simulations

The active core of devices was modelled as a series combination of the doped (TiO*x*) and the undoped (TiO_2_) layers. To account for the coexistence of resistive and capacitive switching, both layers were represented with a parallel of a resistor and a capacitor with distinct resistance and capacitance values for TiO_*x*_ (*R*_ON_ =1 KΩ, *C*_ON_ =0.3 pF) and TiO_2_ layers (*R*_OFF_ =100 KΩ, *C*_OFF_ =0.05 pF). With external bias, the boundary between TiO_*x*_, which has a thickness *ω*(*t*), and TiO_2_ will drift within the thickness (*D*) of the whole device. In other words, the normalised state variable *x*(*t*) (*ω*(*t*)/*D*) will switch within [0, 1]. Considering the drift of the interface between two layers is non-linear and threshold based, *x*(*t*) was assumed to follow a square-wave-like pattern. Then, the overall device resistance and capacitance are calculated based on the assumed series-equivalent circuit structure as *R*(*t*) = *R*_ON_ × *x*(*t*) + *R*_OFF_ × (1 − *x*(*t*)), $C\left(t\right)=\frac{C_{\mathrm{ON}}\times x\times C_{\mathrm{OFF}}\times \left(1-x\right)}{C_{\mathrm{ON}}\times x+C_{\mathrm{OFF}}\times \left(1-x\right)}$. The model was established on Matlab R2012b.

## Results and discussion

**Figure 1:**
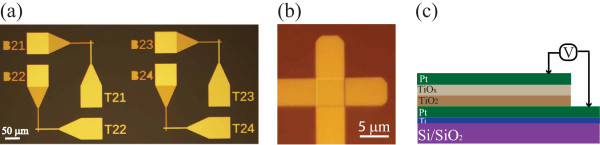
**Optical microscope image, active area, and schematic of different devices. (a)** Optical microscope image of a dense stand-alone device; **(b)** active area of a single 5 × 5 μm^2^ device; **(c)** schematic configuration of the Pt/TiO_2_/TiO_*x*_/Pt devices.

The TiO_2_-based dual active layer devices consist of a semi-conducting TiO_*x*_ layer on top of an insulating TiO_2_ layer, each accessible via a dedicated Pt electrode. Figure 1a illustrates an optical microscope image (top view) of our prototypes with Figure 1b showing a zoom-in image of a single device whose active core occupies an area of 5 × 5 μm^2^. The schematic cross section of the tested devices is depicted in Figure 1c.

**Figure 2:**
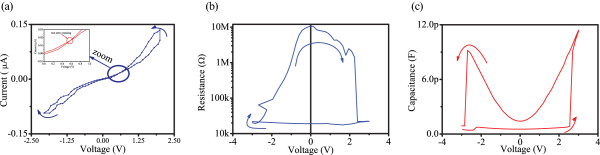
**Resistive and capacitive switching characteristics of Pt/TiO**_**2**_**/TiO**_**x**_**/Pt cells. (a)** non-zero crossing I-V characteristic indicating capacitive capabilities, **(b)** R-V characteristics of TiO_2_ cell, **(c)** matching C-V characteristics.

The *I*-*V* characteristics of a single device (Figure 1b) was first evaluated via DC sweeping measurements with the results presented in Figure 2a. During measurements, the compliance current was maintained at 1 mA to avoid any destructive process (chemical and/or thermal) of the device. For many metal oxide systems, an electroforming step is a prerequisite to facilitate resistive switching [37]. Although the electroforming process exhibits few negative effects, such as required potentials that are substantially larger from the low-voltage devices' thresholds exhibited by CMOS technologies [38], it also creates a variation of switching parameters [39] and a limitation of the device lifetime. In order to avoid these reported issues in this work, all tested devices were electrically characterised without any post-treatment electroforming step. Instead of applying one large potential in sweeping mode, we have used sweeping cycles with gradually increasing the range (maximum value) in order to avoid large potentials. For all devices, we initially employ a quasi-static voltage sweep via DC source-measure units (SMU) for acquiring the characteristic pinched hysteresis *I*-*V* loop which is the memristor signature [1]. Figure 2a illustrates the *I*-*V* characteristics when a 5 V bias was swept in steps of 0.1 V within a ±2 V range. Contrary to the typical signature of an ideal memristor, our device exhibited a bipolar switching response with a non-zero crossing that occurred at 0.6 V as shown in the inset of Figure 1a, demonstrating the presence of parasitic capacitance in our device.

We have then evaluated our devices' capacity to act as memcapacitors [40], via C-V tests using multi-frequency CVU. More specifically, throughout our measurements, we exploited the configuration where real and imaginary components are connected in parallel [41]. AC impedance measurements (1 MHz) of the devices were performed with the AC test signal biased with a voltage sweep within the range ±3 V. In this case, the resistance and the capacitance are calculated based on the measured AC impedance and phase. Figure 2b depicts the resistance-voltage (R-V) characteristics of a Pt/TiO_2_/TiO_*x*_/Pt cell where the arrows indicate the switching directions. A bipolar resistive switching operation was observed with a low-resistive state (LRS) and high-resistive state (HRS) respectively attained after the device was biased with positive SET potential of 2.5 V amplitude and negative RESET potential of −3 V. Because the reset did occur with a smooth transition and without a sharp switching, we have considered the value at the end of negative sweeping which is −3 V.

**Figure 3:**
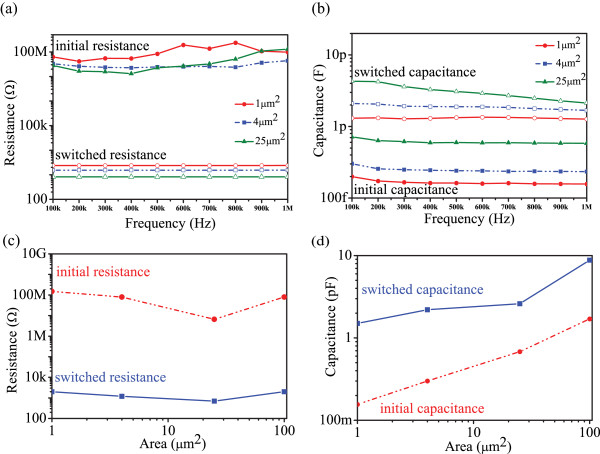
**Frequency and electrode area dependence.** Frequency dependence of **(a)** resistance **(b)** and capacitance. Electrode area dependence of the initial/switched states of the **(c)** resistance and **(d)** capacitance.

On the other hand, a bipolar capacitive switching trends were also observed as demonstrated in Figure 2c. This device is initially in a low-capacitive state (LCS) and a high-capacitive state (HCS) is attained as the voltage bias approaches *V*_SET_ =2.6 V. Reversing the voltage polarity, *V*_RESET_ = −2.7 V causes the device to switch back to LCS. The results indicate that similar SET/RESET potentials are necessary to facilitate both resistive and capacitive switching.Figure 3a,b depicts the frequency dependence (100 kHz to 1 MHz) of the corresponding resistance and capacitance for the initial (pristine) and switched states as a function of devices' active area. These results demonstrate that within this range (100 kHz to 1 MHz), frequency does not play a significant role on either resistive or capacitive states of our devices.

We further investigated the influence of the device's active area on the resistance and capacitance for both pristine (as-fabricated) and switched states. All conductance (*R* =1/*G*) and capacitive measurements were performed via C-V tests by employing 30 mV AC; 1 MHz (DC bias 0.5 V). As shown in Figure 3c, the experimental data do not display any obvious dependence on area, indicating that resistive switching is mainly due to a bulk-based rather than a core/electrode interface mechanism. On the other hand, as expected, the capacitance values for both initial and switched states vary with the electrode areas. It can be observed that small capacitance values (pF range) are defined by a bulk material phenomenon [42,43]. We thus argue that the mechanism responsible for both resistive and capacitive switching is mainly a bulk phenomenon: displacement of the interface between insulating (TiO_2_ region) and semi-conductive (TiO_*x*_) region in the active core facilitated by migration of the ionic species under applied voltage. It is worth pointing out that minimal disturbance of the TiO_2_ stoichiometry such as oxygen vacancies act as n-type dopants or oxygen-excess p-type dopants have a strong impact on the film's resistance and its dielectric properties [44,45]. Various groups have studied the effect of oxygen content on electrical conductivity and demonstrated that the electrical resistance of TiO_2_ is proportional to the concentration of oxygen [44-47]. On the other hand, it was further proven that the same defects (e.g. oxygen vacancies, excess of oxygen) also affect the dielectric constant of TiO_2_[48,49]. On that basis, it can be argued that the TiO_*x*_ (non-stoichiometric) region has a different resistance and capacitance than a TiO_2_ stoichiometric one. However, the resistive and capacitive states are changed when the applied voltage is strong enough to change the oxygen content within the active core and then the insulated (TiO_2_) or doped (TiO*x*) behaviour dominates the overall layer rendering the high or the low resistive and capacitive states.

**Figure 4:**
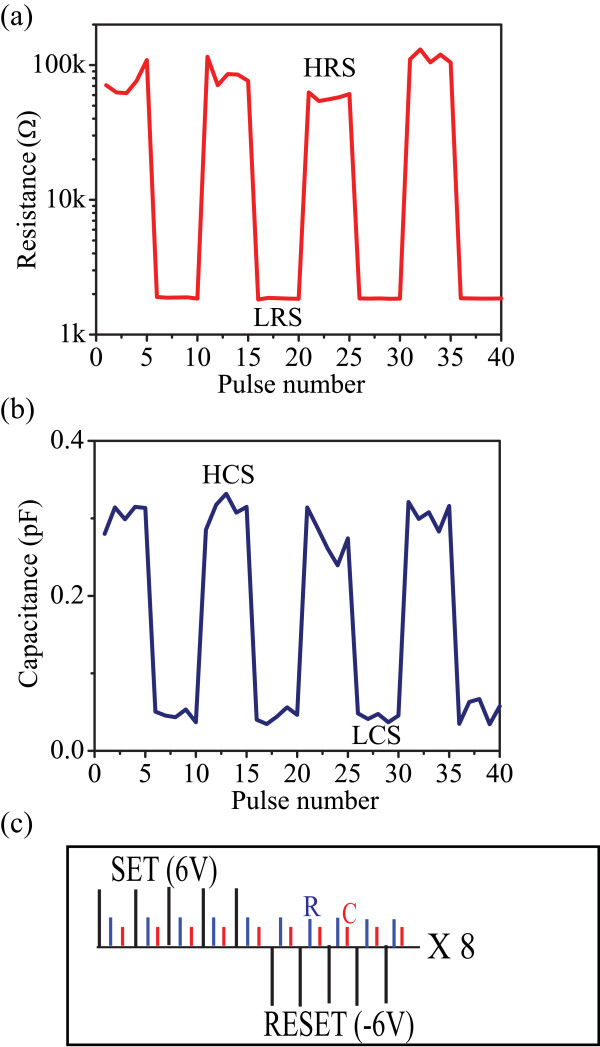
**Concurrent capacitance/resistance modulation of TiO**_**2**_-**based solid-state devices. (a)** Electrical pulse-induced resistance change, **(b)** electric pulse-induced capacitance change, **(c)** pulses sequences utilizing for concurrent resistive and capacitive state programming.

We further investigated the concurrent resistive and capacitive switching in TiO_2_-based cells by employing a voltage-pulsing scheme, as shown in Figure 4c. This bias scheme comprises a train of five SET/RESET voltage pulses of 6 V/1 μs SET and −6 V/1 μs RESET and the full sequence is cycled eight times. After each programming pulse, the resistance is read by applying a positive pulse of 0.5 V and the capacitance is read via C-V tests at 1 MHz via a small super-imposed sensing signal of 30 mV. The pristine state of TiO_2_-based devices was found to be in a HRS and LCS. It should be pointed out that the first positive SET pulse changes the capacitive state from LCS to HCS and the HRS from 100 MΩ to 100 kΩ (not shown here). Nonetheless, persistent application of positive SET pulses induced the changes of the resistive state from HRS to LRS and the capacitive state from HCS to LCS; by applying −6 V RESET pulses, the HRS and HCS states were restored. The exhibited simultaneous capacitive and resistive responses of the device are shown in Figure 4a,b.

Contrary with the results presented in [14], here we observe that the switching trend of resistance is correlated with that of capacitance, i.e. decrease of capacitance with a decrease of resistance. Hence, we argue here that the resistive and the capacitive switching in the investigated devices is mainly induced by the displacement of ionic species through the bulk active core.

**Figure 5:**
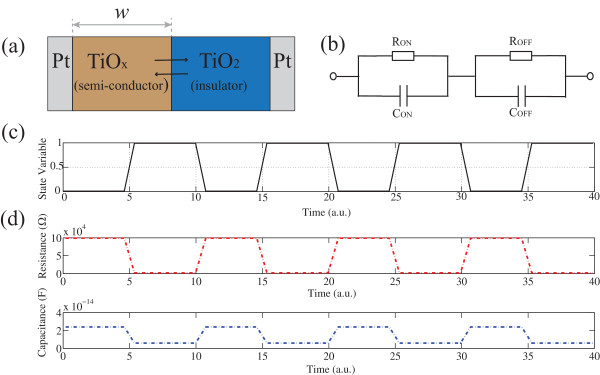
**Schematic, circuit, and results of the proposed model. (a)** Schematic view of our TiO_2_**-**based two-layer devices. **(b)** The equivalent circuit model with two parallel RC elements connected in series. **(c)** Threshold-based dynamics of the state variable *x* (normalised width of the TiO_*x*_ layer). **(d)** Simulated resistive and capacitive switching trends which correlated with experimental results in Figure 4.

Moreover, Figure 5a illustrates the schematic diagram of the proposed model, i.e. displacement of the interface between doped (TiO_*x*_) and undoped (TiO_2_) regions of the active core when the voltage is applied. We propose that both layers could be modelled as a series of two parallel RC elements connected as depicted in Figure 5b. In specific, the semi-conductive TiO_*x*_ layer is represented by a parallel of *R*_ON_ (1 KΩ) and *C*_ON_ (0.3 pF) whilst insulating TiO_2_ layer is modelled with high-value *R*_OFF_ (100 KΩ) and *C*_OFF_ (0.05 pF). Then, we further explored the validity of the proposed equivalent circuit model by evaluating the correlated resistive and capacitive switching with results shown in Figure 5c.

As depicted in Figure 5a, the thickness of the TiO_*x*_ layer is *ω*(*t*), and the total thickness of the device is *D*. To simplify, the normalised state variable is defined as *x*(*t*) = *ω*(*t*)/*D*. To account for the discontinuous switching in Figure 4, we assume that the drift of the interface between two layers is non-linear and threshold based. In other words, the state variable *x* (*ω*(*t*)/*D*) would not arise until the activation energies introduced by the external stimulus exceed the corresponding threshold, as depicted in Figure 5c. In this case, the corresponding resistive and capacitive switching trends were simulated and illustrated in Figure 5d, with the simulated results being in good agreement with the measured ones, in support of our argument on the mechanisms of correlated resistive/capacitive switching.

## Conclusions

In conclusion, by using a complete suite of electrical tests, sweeping (DC and AC) and voltage pulsing, we have shown that memristive and memcapacitive effects emerge naturally in nanoscale TiO_2_ elements with their switching trends being related. The *I*-*V* characteristics of investigated cells display a hysteresis loop with non-zero crossing indicating that our structure also exhibit capacitive effect. Furthermore, the effective resistance and capacitance of TiO_2_/TiO_*x*_ MIM devices were modulated simultaneously by AC sweeping and pulsing modes, rendering resistive and capacitive memory effects in a single device.

## Competing interests

The authors declare that they have no competing interests.

## Authors’ contributions

IS conceived the experiments and performed the electrical characterization. LQ performed the electrical characterization of the samples and simulations. AK fabricated the devices. All authors contributed in the analysis of the results and in the writing of the manuscript. All authors read and approved the final manuscript.
